# How does face mask in COVID-19 pandemic disrupt face learning and recognition in adults with autism spectrum disorder?

**DOI:** 10.1186/s41235-022-00407-4

**Published:** 2022-07-22

**Authors:** Ricky V. Tso, Celine O. Chui, Janet H. Hsiao

**Affiliations:** 1 Department of Psychology & Psychological Assessment and Clinical Research Unit, The Education University of Hong Kong, Hong Kong, SAR China; 2 Department of Psychology, The Education University of Hong Kong, Hong Kong, SAR China; 3 Department of Psychology, The State Key Laboratory of Brain and Cognitive Sciences & Institute of Data Science, The University of Hong Kong, Hong Kong, SAR China

**Keywords:** Autism spectrum disorder, Face masks, Face recognition, COVID-19

## Abstract

Use of face masks is one of the measures adopted by the general community to stop the transmission of disease during this ongoing COVID-19 pandemic. This wide use of face masks has indeed been shown to disrupt day-to-day face recognition. People with autism spectrum disorder (ASD) often have predisposed impairment in face recognition and are expected to be more vulnerable to this disruption in face recognition. Here, we recruited typically developing adult participants and those with ASD, and we measured their non-verbal intelligence, autism spectrum quotient, empathy quotient, and recognition performances of faces with and without a face mask covering the lower halves of the face. When faces were initially learned unobstructed, we showed that participants had a general reduced face recognition performance for masked faces. In contrast, when masked faces were first learned, typically developing adults benefit with an overall advantage in recognizing both masked and unmasked faces; while adults with ASD recognized unmasked faces with a significantly more reduced level of performance than masked faces—this face recognition discrepancy is predicted by a higher level of autistic traits. This paper also discusses how autistic traits influence processing of faces with and without face masks.

## Significance Statement

Face recognition is an integral part of everyday socialization. The disruption of face recognition performance due to the use of face masks will further ignite a socialization challenge for the vulnerable communities, including those with ASD. Indeed, this study uncovered that when faces are learned without a face mask, masking the bottom half of the face generally leads to a lower face recognition performance. In contrast, when faces covered by a facemask are learned initially, typically developing adults benefit with an overall advantage in recognizing both masked and unmasked faces; while adults with ASD recognized unmasked faces with a significantly more reduced level of performance than masked faces—this performance drop is stronger in people with a high level of autistic traits. Face recognition is an integral part of everyday socialization. The disruption of face recognition performance due to the use of face masks will further ignite a socialization challenge for the vulnerable communities, including those with ASD. Our results can spark follow-up studies and attention toward supporting these vulnerable populations during the ongoing COVID-19 pandemic. This is necessary in order to help devise interventions to facilitate face recognition during air-borne pandemics when face masks are used by a vast majority in the general community.

## Introduction

The Coronavirus disease 2019 (COVID-19) pandemic has recently brought abrupt changes to all walks of life. Due to the infectious nature of the disease, various measures have been adopted by various parts of the world to limit its transmission. In places where lockdown has not been imposed, wearing a face mask has been one of such preventive measures widely adopted in highly affected areas (Tso & Cowling, 2020). For example, a survey showed that more that 95% of residents in Hong Kong adopted an everyday practice of wearing face masks in public vehicles and space, a practice which has been made mandatory by law since July 2020 (Tam et al., 2020).

The use of face masks is expected to bring changes to our day-to-day cognition, one of which is the recognition of faces. Face recognition involves the identification of the integrated information from facial features and configuration, a process which has been shown to be holistic (Richler et al., 2011; Hsiao & Galmar, 2016; Chung et al., 2018). Ordinary observers saccade toward both the eyes and the center of a face stimulus, a process in which all the facial features and configural information can be effectively integrated and processed as a whole (e.g., Hsiao & Cottrell, 2008; Chuk et al., 2014; Chuk, Chan, et al., 2017a, Chuk, Crookes, et al., 2017b). These skills have been shown to predict face recognition performances (Hsiao & Liu, 2012; An & Hsiao, 2021; Chuk, Chan, et al., 2017a, Chuk, Crookes, et al., 2017b; Hsiao et al., 2021). Intuitively, the ability to recognize a previously seen face is decreased when the lower half of the face is covered—in the case of the use of face masks by over 90% of the population, everyday face recognition processes are expected to be affected (Freud et al., 2020). As face recognition ability is an integral part of everyday social interaction, social functioning is expected to be disrupted when face recognition is hindered (Tanaka & Sung, 2016).

People with autism spectrum disorder (ASD) are characterized by clinical impairments in socialization and communication, in the presence of restricted patterns of behavior/interests (see *DSM-5;* American Psychiatric Association, 2013). People with ASD are characterized by an increase in eye-contact avoidance and impaired global/holistic processing, all of which are associated with deficits in recognizing face and facial expressions (Halliday et al., 2014; Klin et al., 2002; Tanaka & Sung, 2016). Compared with typically developing adults, people with ASD likely saccade more on the nose and mouth areas of a face during face recognition (Tanaka & Sung, 2016). Hence, those with ASD are likely more vulnerable to the impact of mask use on face recognition when the sight of lower halves of faces are obstructed. Further disruption of face recognition is anticipated in individuals with ASD, who are expected to be impacted profoundly on social outcomes (Tanaka & Sung, 2016). As the majority of the population wear face masks under the COVID-19 pandemic, the extent to which face recognition in people with ASD are affected has not been documented. Hence, one of the aims of this paper is to uncover the effects of mask-covered faces on face recognition performance in people with and without ASD.

Moreover, people with ASD consistently score quantitatively higher than typically developing adults in the Autism Spectrum Quotient (AQ; Baron-Cohen et al., 2001), a self-report inventory that measures autistic traits. A high level of autistic traits is associated with reduced holistic/increased in analytic processing skills, which are reported to predict face recognition performance (Tanaka et al., 2003). Hence, we also measured AQ in participants with and without ASD in order to investigate its association with recognition performance of masked faces in the participants. We hypothesize that participants with higher AQ will be more negatively affected by mask use in the recognition of masked faces.

## Study 1

Study 1 examines the effect of a masked face on face recognition in people with and without ASD, when faces are initially remembered without a face mask.

### Study 1 methods

#### Participants

Sixty-one College students and graduates aged 18 to 26 were invited to participate in the study^1^ (12 females), 29 of which had been diagnosed with ASD by a qualified psychiatric professional prior to participating in this study (6 of which were females). They were recruited from the eight government-funded universities in Hong Kong through the special needs support personnel of each university for clinical assessments during the period from February 2020 to November 2021. Ethical Approval was granted by the Human Research Ethics Committee of our universities for this study.

#### Materials and procedures

*Autism Spectrum Quotient*: At the start of the study, all participants completed the Chinese and Hong Kong-normed version of the Autism Spectrum Quotient (AQ-Adult-HK; see Baron-Cohen et al., 2001; Chan et al., 2018; Poon et al., 2020). With a Cronbach’s alpha coefficients of 0.79 in Poon et al.’s (2020) study, AQ-Adult-HK is a 50-item self-report inventory with items that measures behavioral and cognitive patterns that are often associated with ASD—including social skills, attention switching, attention to detail, communication and imagination. The AQ scores collected were analyzed to examine the association of autistic traits with face recognition performance due to masks across the participants. Moreover, there are no locally-normed standardized diagnostic tools or protocols in Hong Kong for ASD. Therefore, the diagnostic tools used by different psychiatric professionals may vary, though the diagnostic assessment generally involves an array of assessment methods including interviews and psychological testing of behavioral, social and cognitive functioning. Hence, this study administered AQ as a screening tool to all the participants to confirm the difference in the level of autistic quotient between the groups.

##### Non-verbal intelligence

To control for non-verbal intelligence, the nine-item version of the Raven’s Standard Progressive Matrix was administered to each participant. See Bilker et al. (2012) for the psychometric properties.

##### Face recognition performance of masked and unmasked faces

**Fig. 1:**
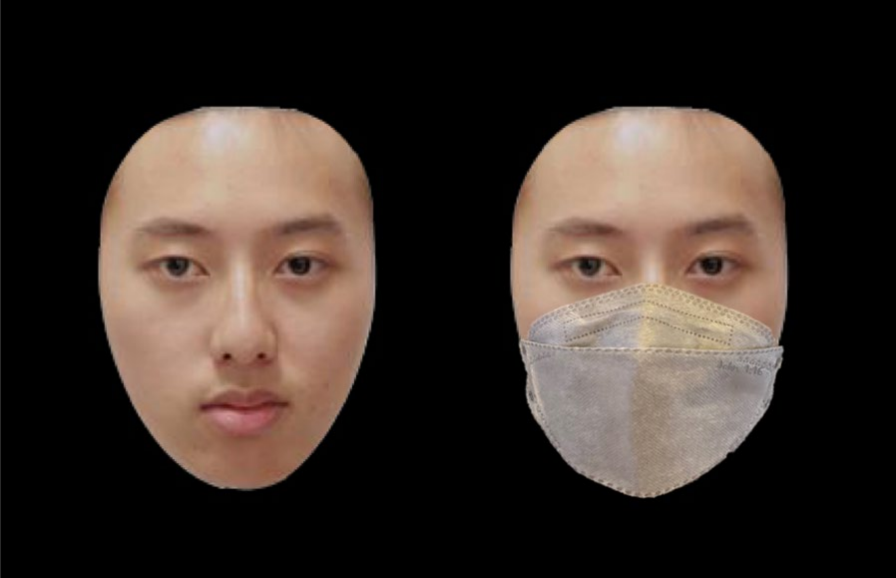
Examples of an unmasked (left) and masked face stimuli

To measure face recognition performance, face stimuli was adopted from the Face Memory subtest in the Weschler Memory Scale–Third Edition (Chinese Version) [WMS-III(Chinese)] (Shu, 2003). The face stimuli used either did not have a face mask (unmasked faces) or had a face mask covering the nose and lower part of each face (masked faces). In this subtest, participants first memorized 20 face stimuli in the learning phase, with each face displayed for two seconds on the computer screen. In the recognition phase, the original Face Memory subtest required participants to view 40 faces, and they responded by reporting orally if they had seen the faces presented to them in the learning phase. In this study’s adaptation, each face stimulus in the recognition phase was presented to each participant twice in a random order, once at its original state (without a face mask), and once with a face mask image covering the lower half of the face. Participants’ response for each trial was recorded by the examiner, with each trial lasting until a response was made. Their discrimination sensitivity A’ for stimuli in the masked and unmasked conditions was measured as follows:

$$A^{\prime} = 0.5 + \left[ {sign(H - F)\frac{{(H - F)^{2} + \left| {H + F} \right|}}{4\max (H,\;F) - 4HF}} \right]$$

where “H” represents the hit rate and “F” the false alarm rate. A′ was used as it is a bias-free nonparametric measure of sensitivity (Liu et al., 2016; Tso et al., 2020, 2021, 2022). See Fig. 1.

### Study 1 results

WMS-III(Chinese) confirmed that all participants had normal face recognition memory. Separate t-tests examined the effect of group (ASD vs. control) on each non-verbal IQ, AQ and EQ. In the nine-item Raven’s test, the ASD and control participants did not differ, t(1, 59) < 1.239, *p* = 0.220, suggesting that both groups had a similar level of non-verbal intelligence. Nevertheless, typical participants scored lower in AQ, t(1, 59) = 8.003, *p* < 0.001, suggesting that participants with ASD generally had a higher level of autistic traits than their typical counterpart. See Table 1 for a summary.

**Table 1:** Summary of AQ, EQ and 9-item Raven’s scores in control and participants with ASD

	ASD Mean (SE)	Control Mean (SE)
Autism Spectrum Quotient (AQ)	135.81 (2.78)	110.00 (1.65)
Non-verbal IQ (9-item Raven’s)	7.52 (0.22)	7.13 (0.22)

**Fig. 2:**
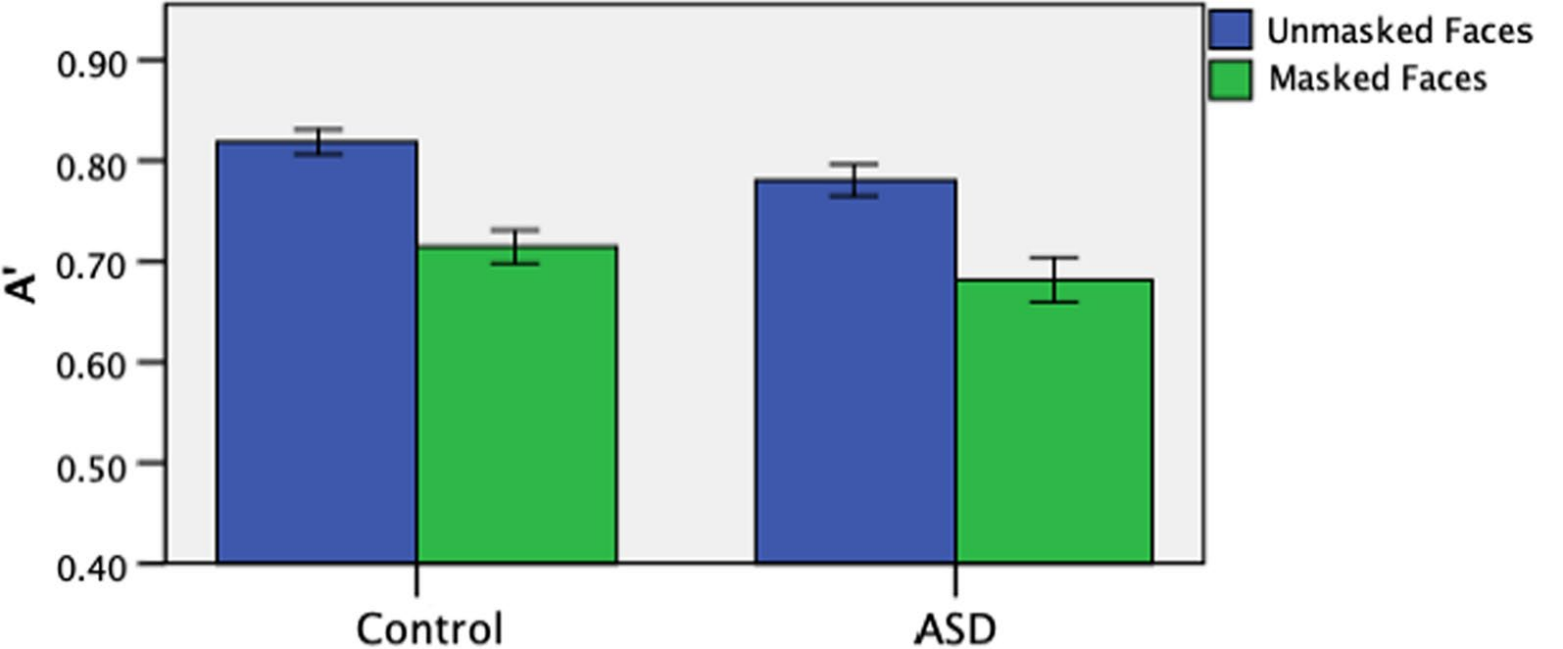
A' in the recognition of unmasked and masked faces in participants with and without ASD

We next examined the performance in recognizing masked and unmasked faces, in which the faces were initially learned without a face mask. We first conducted a 2 (face-masking: masked vs unmasked faces) × 2 (group: ASD vs. control) repeated-measures ANOVA on A', which showed a main effect of face-masking, F(1, 59) = 87.911, *p* < 0.001, η*p*^2^ = 0.598. However, there was no main effect of group, F(1, 59) = 2.834, *p* = 0.098, nor interaction between group and face-masking, F(1, 59) = 0.058, *p* = 0.811 (See Fig. 2).

**Fig. 3:**
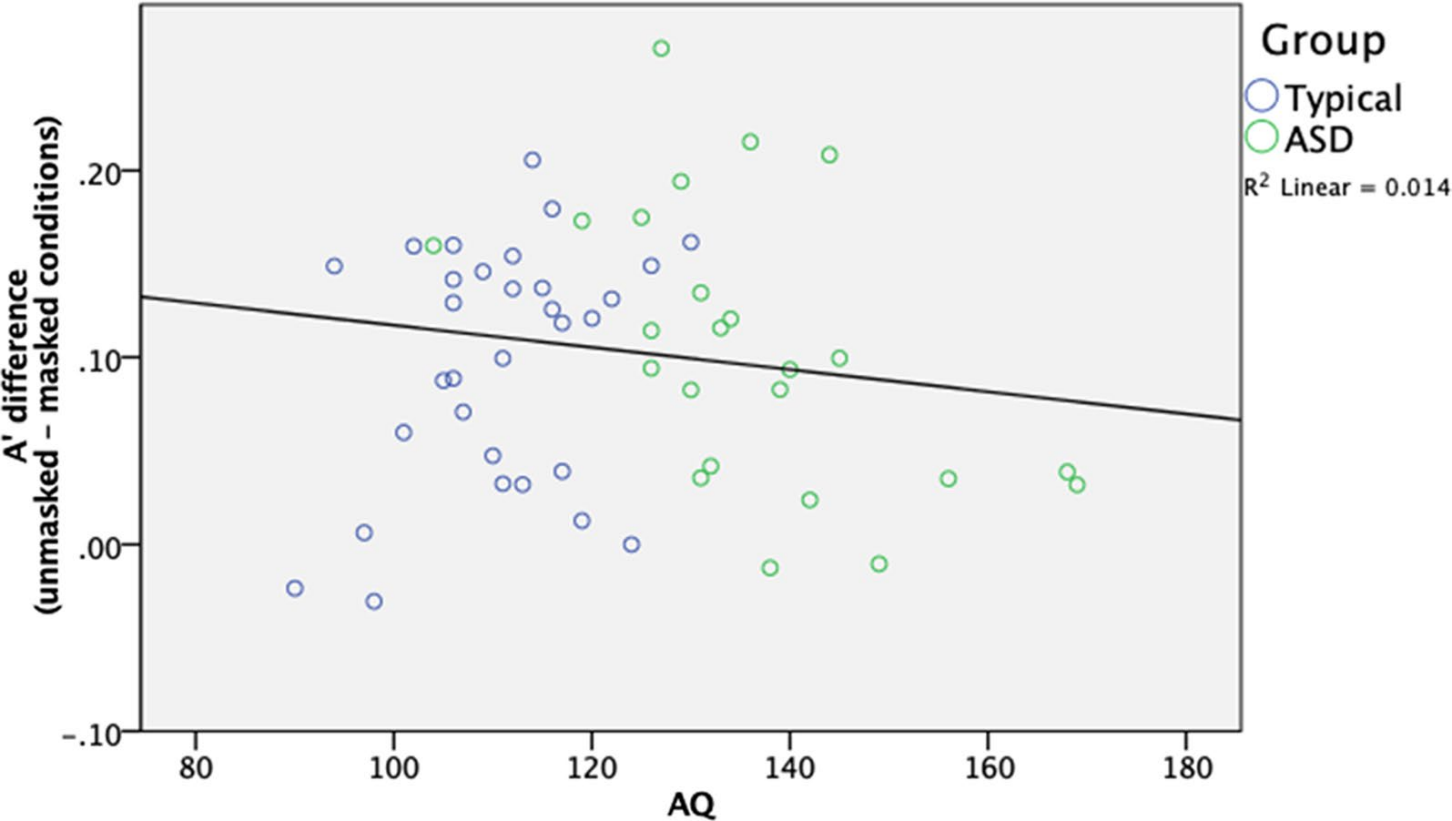
Correlations between A' difference between the unmasked and masked conditions and AQ

Post-hoc analyses showed that participants generally had higher A’ in the unmasked faces condition than in the masked faces condition, t(61) = 9.474, *p* < 0.001, d = 1.213. An additional analysis was included to analyze the reduced performance of face-masking by taking the performance difference between the A' measures in the unmasked and masked conditions (unmasked–masked) for each participant. Pearson’s correlation regression analyses on the entire sample show that this performance difference did not correlate significantly with AQ score, r(58) = 0.119, *p* = 0.374 (see Fig. 3). Overall, covering the lower halves of faces generally lowered face recognition performance when faces were initially learned without a face mask, regardless of the participants’ level of autistic features.

## Study 2

Study 1 simulated the real-life situation in which one is required to recognize masked and unmasked faces that had been initially learned without a face mask, which showed that people with and without ASD had similar levels of performance drop. However, as face masks are increasingly worn by the general community as a daily necessity for a prolonged period, it is often the case when new faces are first learned with a face mask covering the lower part of the face. Hence Study 2 investigated the effect of recognizing masked and unmasked faces in conditions when learned faces are initially masked.

### Study 2 methods

#### Materials and procedures

The same participants from Study 1 were invited to complete Study 2. Participants first memorized 10 masked faces in the learning phase, with each face stimulus displayed for two seconds on the computer screen. In the recognition phase, participants viewed 20 faces, and they responded by reporting orally whether they had seen the faces presented to them in the learning phase. Each face stimulus in the recognition phase was presented to each participant twice in a random order, once at its original unmasked state, and once with a face mask image covering the lower half of the face. Participants’ response in each trial was recorded by the examiner, with each trial lasting until a response was made. Note that the number of stimuli used in Study 2 was reduced to shorten the experimental time in order to shorten the contact time between participants and the examiner as a response to our institute’s measures for the COVID-19 outbreak. Moreover, the faces in Study 1 were not used in Study 2 to avoid familiarity effect.

### Study 2 results

**Fig. 4:**
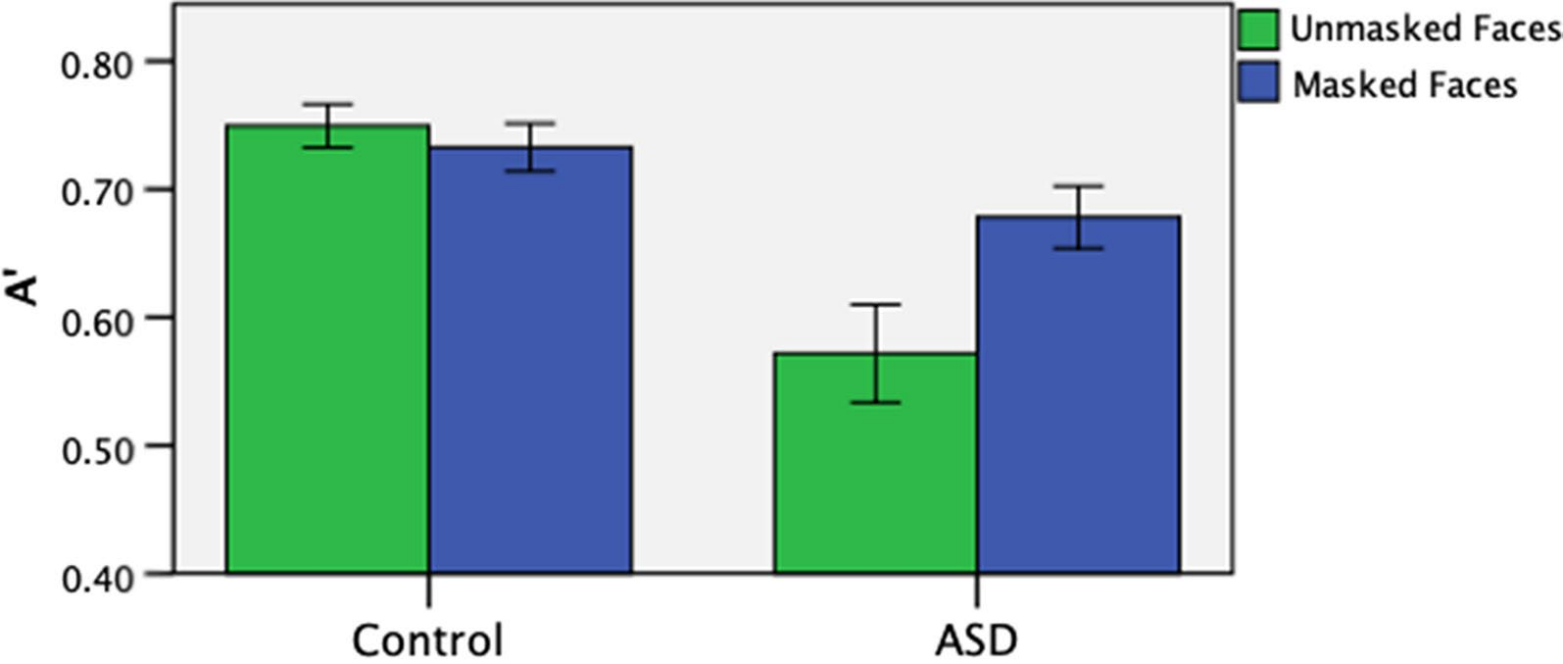
A' in the recognition of unmasked and masked faces in participants with and without ASD

We first examined the recognition performance of masked and unmasked faces. We conducted a 2 (face-masking: masked vs unmasked faces) × 2 (group: ASD vs. control) repeated-measures ANOVA on A', which revealed a main effect of face-masking, F(1, 59) = 5.217, *p* = 0.026, η*p*^2^ = 0.081. There was also a main effect of group, F(1, 59) = 15.095, *p* < 0.001, η*p*^2^ = 0.204, and an interaction between group and face-masking, F(1, 59) = 9.843, *p* = 0.003, η*p*^2^ = 0.143. See Fig. 4.

**Fig. 5:**
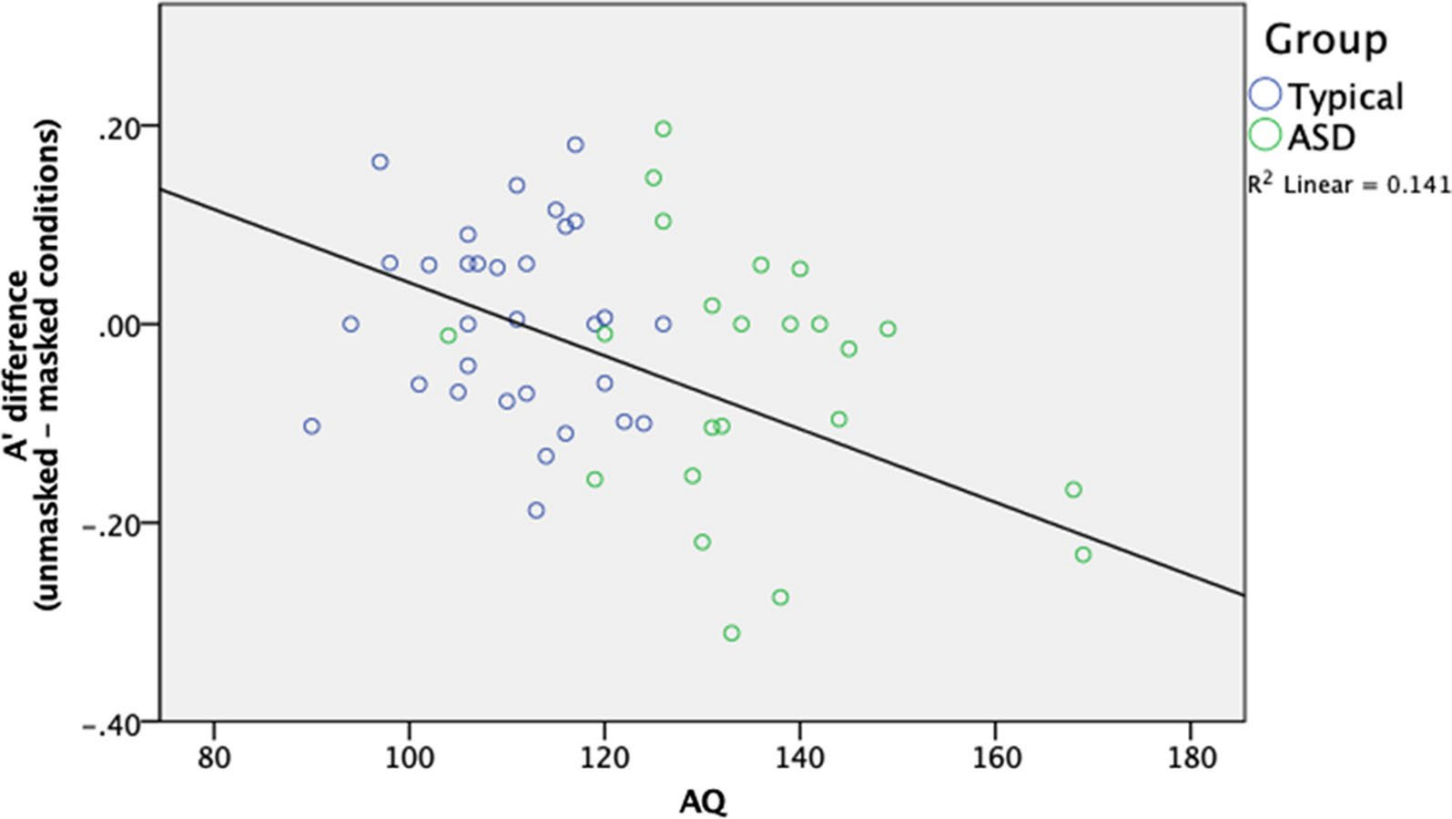
Correlations between difference in A' between the unmasked and masked conditions AQ

Post-hoc analyses showed that while ASD participants performed better in recognizing masked faces than unmasked faces, t(28) = 3.059, *p* = 0.005, d = 0.568, the control participants showed similar performances in recognizing masked and unmasked faces, t(31) = 0.833, *p* = 0.411, d = 0.254. As compared with ASD participants, the control participants performed only marginally better at recognizing masked faces, t(59) = 1.800, *p* = 0.077, though they recognized unmasked faces significantly better than participants with ASD, t(59) = 4.389, *p* < 0.001, d = 1.107. Pearson’s correlation regression analyses on the entire sample showed that the performance difference between the A' measures in the unmasked and masked conditions (unmasked – masked) correlated significantly with AQ score, r(58) =  −0.375, *p* = 0.004 (see Fig. 5; note that the scores were centralized).

## Discussions

When faces were first learned without a face mask, Study 1 showed a general poorer performance in the recognition of masked faces regardless of whether participants had a diagnosis of ASD. In contrast, Study 2 showed that when faces covered by a face mask were initially learned, recognition performance of unmasked faces was lower compared with recognizing masked faces in ASD participants, whereas typical participants had a similar level of recognition performance for masked and unmasked faces. In this section, we discuss the observed patterns in this paper according to the current literature on face recognition in individuals with ASD and masked face recognition.

### Cognitive flexibility in shifting recognition strategies

In Study 2, we found that individuals with ASD had poorer performance than typical participants in the recognition of unmasked faces that were learned with a masked on. Hsiao et al. (2022) showed that in this scenario, typical adults who adjusted their face-recognition strategies by shifting their gazes toward the eyes of the unmasked faces had better recognition performance. This shift in eye gaze strategy toward the eyes is beneficial in this scenario since these unmasked faces were learned with a mask on, revealing only the information around the eye region for memory encoding. Thus, during memory retrieval, only the information around the eye region is informative. This shift in recognition strategies is likely to involve cognitive flexibility. Indeed, reduced cognitive flexibility has long been established as a hallmark of ASD (de Vries & Geurts, 2012; Halliday et al., 2014; Klin et al., 2002; Tanaka & Sung, 2016; Van Eylen et al., 2011). Since people with ASD generally have reduced cognitive flexibility (de Vries & Geurts, 2012; Van Eylen et al., 2011), perhaps their recognition performance of unmasked faces that were learned with a mask on were poorer than typical adults due to a reduced ability to shift their recognition strategies to match what was encoded during face learning, that is, to shift gaze toward the eyes instead of continuing using their original strategy for unmasked faces. More specifically, although both ASD and typical adults were likely to fixate the eye region during face learning since it was the only face region available during learning of masked faces, participants with ASD likely continued making saccades to the nose and mouth areas during face recognition of unmasked faces more often than typical adults (Tanaka & Sung, 2016), resulting in worse performance. Another possibility was impaired interference control in ASD (Geurts et al., 2014): Since recognizing an unmasked face learned with a masked on was the only condition where more facial information was available during recognition than during learning, participants with ASD might exhibit greater vulnerability to distractions by task-irrelevant facial features (i.e., nose and mouth regions). Consistent with the above speculations, we found that participants’ AQ was negatively correlated with the performance difference between recognizing an unmasked face and recognizing a masked face when the face was learned with a mask on: the higher the AQ, the worse the performance in recognizing an unmasked face relative to recognizing a masked face.

In contrast, in Study 2, when recognizing masked faces that were also learned with a mask on, participants with ASD did not perform significantly differently from the typical group. Similarly in Study 1, when recognizing unmasked faces that were also learned without a mask, the two participant groups did not differ in recognition performance. These results suggested that when mask conditions during face learning and face recognition matched and thus eye gaze strategies engaged during face learning did not need to be adjusted during recognition, the two participant groups did not differ in performance. This result was consistent with our speculation that the poorer performance in the recognition of unmasked faces that were learned with a mask on in the ASD than the typical group may be related to reduced cognitive flexibility typically observed in individuals with ASD.

Note however that in Study 1, when recognizing masked faces that were learned without a mask, the ASD and typical groups did not differ in performance, although mask conditions during learning and recognition did not match. We speculated that in this scenario, both the ASD and typical groups would look at the eye region during the recognition of masked faces since the nose and mouth regions, although were available for viewing during face learning, were unavailable during recognition. In other words, their strategy change during recognition would be mainly guided by the mask and rely less on cognitive flexibility or interference control, and thus no significant performance difference was observed between the two participant groups. Hsiao et al. (2022) showed that in this scenario, typical adults whose eye fixation behavior was more consistent across trials had better performance, and this eye fixation behavior consistency was particularly correlated with non-verbal IQ as measured in the nine-item Raven’s test. Since in the current study the ASD and typical groups were matched in non-verbal IQ, the result that they did not differ in performance in this scenario was consistent with Hsiao et al.’s finding (2022). Note however that although IQ is related to executive functioning, ASD individuals may still differ in their executive functioning skills from the typical adults when IQ was matched (Benallie et al., 2021), and this potential executive functioning difference might be related to the performance difference observed in Study 2 when recognizing unmasked faces that were learned with a mask on. Future work may examine this possibility. Together these results also suggest that different cognitive abilities were required in these challenging scenarios where the mask conditions during face learning and recognition do not match, and individuals with ASD may be particularly vulnerable in the scenario when a face learned with a mask on has to be recognized without a mask.

## Limitations

Note that AQ does not specifically measure cognitive flexibility nor eye movement. Therefore, the speculations of the association of AQ with the results in the paper must be interpreted with cautions. Future studies should employ objective measurements of eye movements and cognitive skills in order to examine the unique contribution of eye movement behavior and cognitive inflexibility on masked-face learning and recognition.

Another point to note is the small sample size as a result of disruption of the study due to the COVID-19 outbreak. Our challenge in recruitment of adult college participants with ASD is exacerbated by the under-enrollment of students with ASD in Hong Kong colleges, as well as under-diagnosis of ASD from the cohort in the age group. Future studies should expand the recruitment of people with ASD across different age groups and backgrounds in order to increase the sample size.

## Conclusion

To summarize, results from Study 1 suggest that when faces were learned without a face mask, masking the bottom half of the face generally leads to a lower face recognition performance in both adults with and without ASD. In contrast, Study 2 suggests that autistic traits predict learning of masked faces. Results from Study 2 hint at the importance of cognitive flexibility in learning new masked faces, in line with previous studies that found an association between face recognition and autistic traits (Tanaka & Sung, 2016; Tanaka et al., 2003). Our results hence contribute to the literature on our understanding of face recognition in people with autistic traits in different situations when they are required to recognize or learn to identify faces that are covered by a face mask. Future studies using eye-tracking or paradigms that measure holistic processing and cognitive flexibility can allow us to identify the exact underlying cognitive mechanisms on the effect of face masks on face recognition. This is necessary in order to help devise interventions to facilitate face recognition during air-borne pandemics when face masks are used by a vast majority in the general community.

## Data Availability

The datasets of the current study are available from R.V. Tso the corresponding author on reasonable request.

## Abbreviations

AQ: Autism spectrum quotient
ASD: Autism spectrum disorder
COVID-19: Coronavirus disease 2019
EQ: Empathy Quotient
WMS-III: Weschler Memory Scale–Third Edition

## Footnotes

1 According to a power analysis, to detect a within-between interaction, assuming a medium effect size f = 0.25, alpha = 0.05, power = 0.8, the required sample size is 34.
